# Effect of agomelatine on memory deficits and hippocampal gene expression induced by chronic social defeat stress in mice

**DOI:** 10.1038/srep45907

**Published:** 2017-04-04

**Authors:** Vincent Martin, Najib Allaïli, Marine Euvrard, Tevrasamy Marday, Armance Riffaud, Bernard Franc, Elisabeth Mocaër, Cecilia Gabriel, Philippe Fossati, Stéphane Lehericy, Laurence Lanfumey

**Affiliations:** 1 Centre de Psychiatrie et Neurosciences, INSERM UMR 894, Université Paris Descartes, Paris, France; 2 Centre de NeuroImagerie de Recherche - CENIR– Inserm UMR1127- CNRS 7225, Institut Cerveau Moelle - ICM, Sorbonne Universités, UPMC UMR S 1127, Paris, France; 3 Institut de Recherches Internationales Servier, IRIS, Suresnes, France; 4 Social and Affective Neuroscience - SAN Laboratory - Inserm U 1127- CNRS UMR 7225- Institut du Cerveau et de la Moelle- ICM - Sorbonne Universités, UPMC UMR S 1127, Paris, France

## Abstract

Chronic stress is known to induce not only anxiety and depressive-like phenotypes in mice but also cognitive impairments, for which the action of classical antidepressant compounds remains unsatisfactory. In this context, we investigated the effects of chronic social defeat stress (CSDS) on anxiety-, social- and cognitive-related behaviors, as well as hippocampal *Bdnf*, synaptic plasticity markers (*PSD-95, Synaptophysin, Spinophilin, Synapsin I* and *MAP-2*), and epigenetic modifying enzymes (*MYST2, HDAC2, HDAC6, MLL3, KDM5B, DNMT3B, GADD45B*) gene expression in C57BL/6J mice. CSDS for 10 days provoked long-lasting anxious-like phenotype in the open field and episodic memory deficits in the novel object recognition test. While total *Bdnf* mRNA level was unchanged, *Bdnf* exon IV, *MAP-2, HDAC2, HDAC6* and *MLL3* gene expression was significantly decreased in the CSDS mouse hippocampus. In CSDS mice treated 3 weeks with 50 mg/kg/d agomelatine, an antidepressant with melatonergic receptor agonist and 5-HT_2C_ receptor antagonist properties, the anxious-like phenotype was not reversed, but the treatment successfully prevented the cognitive impairments and hippocampal gene expression modifications. Altogether, these data evidenced that, in mice, agomelatine was effective in alleviating stress-induced altered cognitive functions, possibly through a mechanism involving BDNF signaling, synaptic plasticity and epigenetic remodeling.

Chronic social stress, which in humans often originates from social relationships encountered throughout the entire life^1^,^2^, has been proposed to be a major cause for depression^3^. Stress can lead to multiple psychological and neurobiological disorders^4^ such as cognitive deficits often associated with depression^5^. In many cases, in addition to emotional-related symptoms, depressed patients also suffer from cognitive problems including memory loss, attentional impairment, executive dysfunction and poor decision making. However, and despite the different families of antidepressant compounds available to treat depressive symptoms, cognitive deficits are still not adequately alleviated by regular antidepressant therapies^6^,^7^.

Social stress can easily be transposed to rodents, as their society life organization is based on a clearly identified social hierarchy^8^. To this aim, the chronic social defeat stress (CSDS) paradigm has been developed and validated to determine the behavioral and molecular effects of social stress^9^,^10^. This stress is based on repeated subordination to unfamiliar dominant in its own territory. Following repeated exposures to aggressive resident CD-1 mice, intruder C57BL/6J mice display various behavioral deficits such as social avoidance, anxiety-like phenotype, despair-like behavior and a reduction in sweet preference^11^,^12^,^13^. In addition, CSDS-exposed animals also exhibit cognitive impairments^14^,^15^,^16^, related to those observed in patients subjected to social stress, that suggest a marked dysregulation of the hippocampus, a brain structure known to play a crucial role in memory processes^17^, and to be directly altered by social stress^18^.

The effect of antidepressant drugs on memory alterations has been assessed on several chronic stress models, but their efficacy is still a matter of debate^19^,^20^,^21^,^22^. Among the different antidepressant compounds, agomelatine is an agonist at melatonergic MT1 and MT2 receptors and a 5-HT_2C_ receptor antagonist displaying antidepressant and anxiolytic properties in several validated animal models. Agomelatine is also able to modulate neuroplasticity in both basal conditions and in models of depression (see refs 23,24 for review).

Moreover, agomelatine at antidepressant doses has been shown to display beneficial effects on memory in naïve animals after acute^25^ and chronic treatment^26^ in the rat NOR test. Another study also reported that agomelatine was able to reverse the memory impairments provoked by chronic mild stress in mice as assessed in the NOR test and the Morris water maze^27^. In this line, agomelatine was also shown to block memory impairment induced by a stress in rats trained in a spatial memory task^28^ and to improve social memory in prenatal stressed rats^29^. Recently, we demonstrated that chronic agomelatine could prevent hippocampal epigenetic modifications that occurred in mice following chronic ultra-mild stress protocol, suggesting that agomelatine could achieve its mechanism of action through epigenetic remodeling^30^. In order to further explore the consequences of CSDS on anxiety, social behavior and memory functions, as well as hippocampal gene expression, we performed a series of molecular and behavioral experiments and showed that the consequences of such a social stress, in particular those related to cognition, could be reversed by chronic agomelatine treatment in mice.

## Materials and Methods

### Animals and treatments

Adult male C57BL/6J mice (7 week-old; Charles River Laboratories, l’Abresle, France) were housed 4–6 per cage under standard laboratory conditions (22 ± 1 °C, 60% humidity, 12-h light-dark cycle with lights on at 07:00, food and water *ad libitum*) for one week before starting experiments. All procedures concerning animal care and treatment were carried out in accordance with the protocols approved by the ethical committee # C2EA -05 Charles Darwin for the use of experimental animals and were licensed by the Directorate General for Research and Innovation (French Ministère de l’Enseignement Supérieur et de la Recherche), under protocol authorization # 00966.02.

Agomelatine (50 mg/kg/day, Servier Laboratories, Suresnes, France) or its vehicle HEC (hydroxyethylcellulose 1%, Servier Laboratories) were administered intraperitoneally (i.p.) on a daily basis for 21 days (days 11–31), 2 hours before the dark phase.

### Chronic social defeat stress (CSDS)

Chronic social defeat stress was performed as previously described^9^,^10^. Male CD-1 retired breeder mice were screened for aggressive behavioral response measured by the latency to initial aggression that should be less than 60 s in at least two consecutive 180 s-screening sessions^31^. 24 h before the first defeat, mice were singly housed in one side of a standard cage separated by a clear perforated Plexiglas divider, which allowed sensory but not physical contact. An experimental C57BL/6J intruder mouse was exposed to a CD-1 aggressor for 10 min, during which the intruder mouse was attacked and displayed subordinate posturing. To avoid physical injury, mice were briefly isolated in case of over-aggressive behavior from the CD-1. After a 10-min defeat session, the experimental mouse was placed for the rest of the day on the opposite side of the divider. This procedure was repeated for 10 consecutive days (D1–D10), using a different aggressor CD-1 mouse every day, between 10:00 and 11:00 am. Control mice were housed by pair in a similar cage, separated by a clear Plexiglas divider, and never exposed to CD-1 mice. Immediately after the last defeat session, stressed and control mice were singly housed in a new standard cage.

In order to assess the effect of chronic agomelatine treatment on stress-induced behavioral deficits, susceptible mice were divided into two homogenous groups: “stressed-AGO” that included stressed mice treated for 3 weeks with agomelatine (50 mg/kg/day, i.p.) and “stressed-HEC” that included stressed mice treated with vehicle (HEC) in the same conditions. Control mice treated for 3 weeks with HEC were called “control-HEC”. Mice were weighted once a week in order to monitor body weight gain. Neither stress nor treatments significantly modified body weight (See Table S1), although there is a trend for a higher increase in weight after social stress compared to controls which actually fits with previous data on the leptin resistance-induced by social stress^32^.

### Behavioral testing

**Figure 1:**
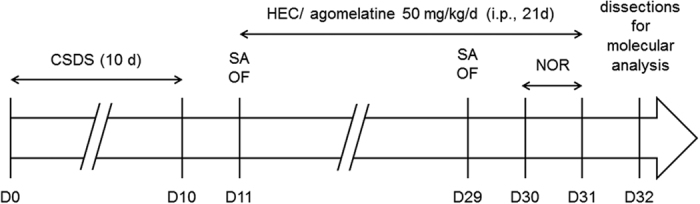
Study design. Mice were submitted to CSDS for 10 days and then treated 21 days with either agomelatine or its vehicle HEC i.p. Anxiety- and depressive-like phenotypes were assessed at day 11 and day 29 using the social avoidance and open field test, and memory was tested using the novel object recognition test at days 30 and 31. Finally, animals were sacrificed at day 32 for molecular analysis. CSDS, chronic social defeat stress; HEC, hydroxyethylcellulose; NOR, novel object recognition test; OF, open field; SA, social avoidance test.

On D11, defeated and control animals were tested in the open field and social avoidance tests, in order to select the susceptible, which displayed anxiety- and social avoidance phenotypes, and the resilient mice^10^, and agomelatine or HEC chronic treatments were initiated at the same day. Similar behavioral tests were replicated on D29 to assess the effect of treatments. In addition, on D30 and D31, a novel object recognition test was carried out to evaluate the effect of stress and treatments on episodic memory (see Fig. 1).

### Open field test (OF)

24 h after the last defeat session (D11), mouse anxiety levels and locomotor activity were tested in the open field, consisted of four open boxes (50 × 50 cm) separated by Plexiglas walls and equipped with an infrared floor for automatic exploratory behavior detection (View Point, Lyon, France). A virtual zone (20 × 20 cm) was delimited in the center of the open field. Mice were placed in the boxes, and left free to explore for 1 h under low light conditions (5 lux). The number of entries, the time spent at the center and the total distance covered in the entire open field were measured using a video tracking system (Viewpoint).

### Social avoidance test

Social avoidance was assessed three hours after the open field test, as previously described^9^,^10^. The test consisted in two consecutive 150 s-sessions, under low light conditions (5 lux). Mice were subjected to the test in the same open field boxes they were previously habituated during the open field test. In the first session, experimental mice were allowed to explore the open field containing an empty circular wire cage (18 × 9 cm) located at one end of the field. In the second session, conditions were identical except that the circular cage contained a CD-1 aggressive mouse (defined as “target”). A virtual interaction zone (area projecting 8 cm around wire cage) was delimited, and the time spent in this zone was scored during both sessions using a video tracking system (Viewpoint). Social interaction behavior was estimated as interaction ratio: (time spent in the interaction zone in the presence of target/time spent in the interaction zone in the absence of target) * 100. Mice were classified either as susceptible with ratio below 100 or as resilient with ratio above 100 on a social avoidance test performed at D11, as described in the literature^10^. Within the CSDS-subjected group, only susceptible mice were included for the subsequent experiments. No such sorting was performed within the control group.

### Novel object recognition test (NOR)

The test was performed as previously described with slight modifications^33^. The apparatus consisted of a black open arena (30 × 30 cm), with three types of objects (colored plastic cylinders, white plastic boxes and transparent glass bottles). Room light was kept at 5 lux. Mice were trained to the apparatus for 15 min in the morning of D30, and the test was carried out in the afternoons of D30 and D31. During the acquisition phase, two identical objects were placed in the arena, on the right and left corners, at 7 cm-distance from the walls. Mice were introduced in the arena and left free to explore for 5 min, and returned to their home cage. After a 1h-intertrial interval (ITI1), one of the objects was replaced by a different one, and the mice were reintroduced in the arena for another 5 min-session (recognition phase 1, D30). The procedure was replicated 24 h (ITI24) after the first recognition phase, with a different new object (recognition phase 2, D31). In both testing phases, the time spent by the mice exploring the objects (touching with their nose or forelimbs, or sniffing at a distance less than 1 cm) was manually scored from recorded video using XNote StopWatch software. The recognition index was calculated as follow: (time spent exploring the novel object - time spent exploring the familiar object)/(time exploring both novel and identical objects). Mice that explored both objects less than 5 s during recognition phase 1 or 2 were excluded.

### Quantification of RNA levels by quantitative RT-PCR

On D32, at the end of treatment, animals were sacrificed by cervical dislocation and the hippocampus was quickly dissected, frozen in liquid nitrogen and kept at −80 °C for molecular analysis. Total mRNA was extracted using TRI Reagent Solution (Life Technologies, Saint Aubin, France) following manufacturer’s instructions. Reverse transcription was performed with High Capacity cDNA Reverse Transcription kit (Applied Biosystem, Courtaboeuf, France) with the following cycling protocol: 25 °C for 10 min, 37 °C for 2 h and 85 °C for 5 s. cDNA samples were stored at −20 °C. Amplification reactions were performed with KAPA SYBR FAST qPCR Master Mix (Clinisciences, Nanterre, France) following manufacturer’s instructions using the 7300 Real Time PCR System (Applied Biosystem, Courtaboeuf, France). The following cycling protocol was applied: 95 °C for 3 min, followed by 40 cycles of 95 °C for 15 s, 60 °C for 30 s and 72 °C for 30 s. The list of primers used is indicated in Table 1. β-actin was used as housekeeping gene for normalizing gene expression results. The 2^∆∆CT^ (Delta Delta Comparative Threshold) method was used to quantify the fold change in mRNA expression in the different experimental groups.

### Statistical analysis

Data (displayed as mean ± S.E.M.) were analyzed using Prism 5 Software (GraphPad, San Diego, USA). To compare “control-HEC”, “stressed-HEC” and “stressed-AGO” groups, a one-way analysis of variance (ANOVA) was used, followed by Newman Keuls multiple comparisons test. When variances were significantly different, a Kruskal-Wallis test was used, followed by a Dunn’s multiple comparisons tests. For comparing “control” and “susceptible” groups, an unpaired Student’s t-test was used, with Welch’s correction if needed.

## Results

### Effect of CSDS and chronic agomelatine treatment on depression-related behavior in the social avoidance test

**Figure 2:**
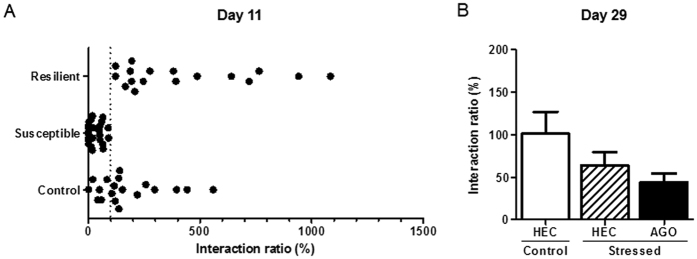
Effect of chronic social defeat stress and chronic agomelatine (50 mg/kg/day i.p.) on social interaction in the social avoidance test. (**A**) The day after the last defeat session (Day 11), mice subjected to CSDS can be split into two groups: the susceptible mice, which display a significant reduction in the interaction ratio compared to control mice, and the resilient mice, which do not behave differently from non-stressed group and display a significant higher interaction ratio than the susceptible mice. (**B**) On Day 29, no significant difference was found between control-, stressed mice treated with HEC and stressed mice treated with agomelatine. Each bar is the mean ± S.E.M. of n = 19 (control mice), 11 (stressed HEC mice) and 11 (stressed AGO mice). AGO, agomelatine; HEC, hydroxyethylcellulose.

The interaction ratio was used to discriminate susceptible and resilient mice to chronic stress among a batch of mice all subjected to CSDS. As shown in Fig. 2A and S1, social avoidance test led to the segregation of susceptible mice (n = 24), and resilient mice (n = 17).

Social avoidance test was then replicated on D29, after a 3-week treatment with either agomelatine or HEC in CSDS-exposed mice, or HEC in control mice (Fig. 2B and S1). Although a Kruskal-Wallis test failed to reveal any overall difference between the three groups of mice (p = 0.16), it can be noted that stress still tended to reduce interaction ratio, but there was no effect of the agomelatine treatment.

### Effect of CSDS and chronic agomelatine treatment on anxiety-related behavior in the open field

**Figure 3:**
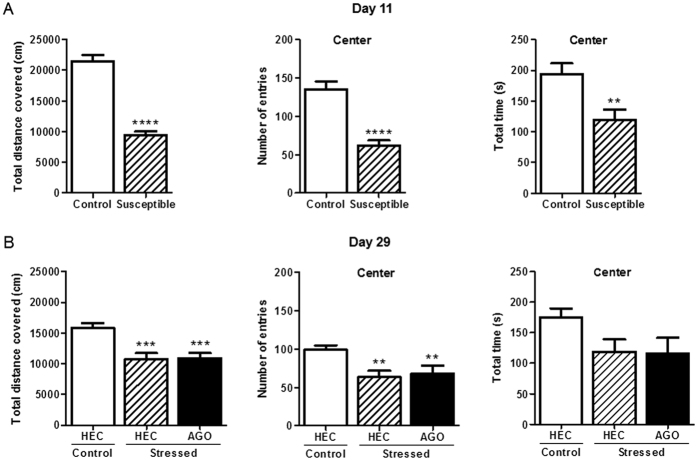
Effect of chronic social defeat stress and chronic agomelatine (50 mg/kg/day i.p.) on anxiety-related behavior in the open field. (**A**) The day after the last defeat session (Day 11), susceptible mice showed a significant hypolocomotion (**A**, left), as well as a reduced exploration in the center of the open field (**A**, middle and right) compared to control mice. **p < 0.01, ****p < 0.0001, “Stressed” vs “Control”, unpaired Student’s t-test with Welch’s correction when needed. Each bar is the mean ± S.E.M. of n = 19 control and 22 susceptible mice. (**B**) On Day 29, stressed mice chronically treated with the vehicle HEC still displayed less exploration in the entire open field and at the center (**B**, left and middle). However, no significant difference was found between the three conditions for the time spent at the center (**B**, right). Chronic agomelatine did not block the effects of stress on the reduced exploratory behavior in the open field (**B**, left and middle). **p < 0.01, ***p < 0.001, “Stressed HEC” or “Stressed AGO” vs “Control HEC”, one-way ANOVA followed by Newman-Keuls multiple comparisons test. Each bar is the mean ± S.E.M. of n = 19 (control mice), 11 (stressed HEC mice) and 11 (stressed AGO mice). AGO, agomelatine; HEC, hydroxyethylcellulose.

In order to assess the effect of CSDS on anxiety-like behavior, mice were subjected to the open field test on D11 (Fig. 3A). Susceptible mice displayed a significant hypolocomotion in the entire open field (p < 0.0001, Student’s t-test with Welch’s correction), and a decreased number of entries (p < 0.0001) and time spent at the center of the open field (p = 0.0007) compared to control mice.

The open field test was repeated on D29, to evaluate the effect of treatments on stress-induced deficits (Fig. 3B). After a 3 week chronic vehicle treatment, one way ANOVA followed by Newman-Keuls multiple comparisons test showed that stressed-HEC mice still showed reduced locomotion [F(2,41) = 5.737, p < 0.001] and entry number in the center of the open field (F(2,41) = 4.461, p < 0.01). Although there was a slightly significant overall difference in the mean time spent at the center between the three groups (p = 0.0479), a Newman Keuls multiple comparisons test failed to reach statistical significance. Chronic agomelatine treatment was not able to prevent stress-induced hypolocomotion and decreased exploration in the center of the open field [F(2,41) = 5.529, p < 0.001; F(2,41) = 3.966, p < 0.01, respectively].

### Effect of CSDS and chronic agomelatine treatment on episodic memory in the novel object recognition test

**Figure 4:**
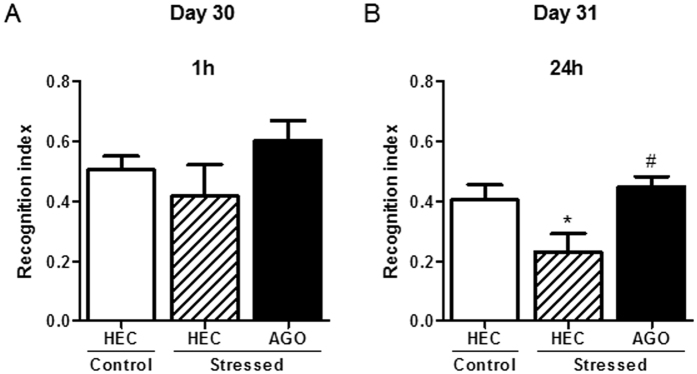
Long-lasting effect of chronic social defeat stress and chronic agomelatine (50 mg/kg/day i.p.) on episodic memory in the novel object recognition test. Mice were tested in the novel object recognition on D30 (A, ITI1) and D31 (B, ITI24). Memory performances were represented by the recognition index. No significant differences were found between the three groups during the recognition phase 1 that occurred 1 h after the acquisition phase (**A**). However, after a 24 h-ITI, stressed-HEC mice discriminated the novel object less than control-HEC mice and the deficit was prevented by chronic agomelatine treatment in stressed-AGO mice (**B**). *p < 0.05 “Stressed HEC” vs “Control HEC”, ^#^p < 0.05 “Stressed AGO” vs “Stressed HEC”, Kruskal-Wallis test followed by Dunn’s multiple comparisons test. Each bar is the mean ± S.E.M. of n = 16 (control mice), 8 (stressed HEC mice) and 10 (stressed AGO mice). AGO, agomelatine; HEC, hydroxyethylcellulose.

Novel object recognition test performed on D30 and D31 was used to determine the effect of CSDS and that of chronic agomelatine on mouse episodic memory. ITI 1 h and 24 h allowed evaluating respectively the short- and long-term memory. In the recognition phase 1 with ITI1, a Kruskal-Wallis test revealed no overall difference between the different groups (p = 0.3377) (Fig. 4A). However, in the recognition phase 2 with ITI24, a one-way ANOVA followed by Newman-Keuls multiple comparisons test showed that stressed-HEC displayed a significantly decreased in the recognition index compared to control-HEC mice [F(2,30) = 3.209, p < 0.05] (Fig. 4B). In contrast, a one-way ANOVA showed that stressed-AGO mice did not significantly differ from control-HEC [F(2,30) = 0.8906, p > 0.05] and that the recognition ratio was significantly increased in comparison to that of stressed-HEC mice [F(2,30) = 3.679, p < 0.05].

### Effect of CSDS and chronic agomelatine treatment on total *Bdnf* and *Bdnf* exons IV and VI mRNA expression

**Figure 5:**
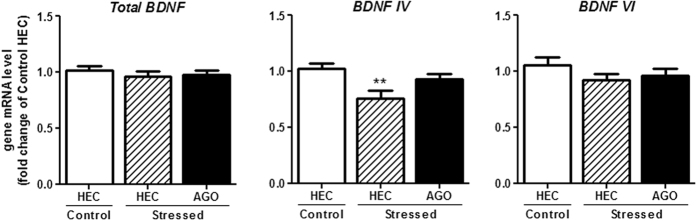
Long-lasting effect of chronic social defeat stress and chronic agomelatine on total *Bdnf* and *Bdnf* exons IV and VI mRNA expression in the hippocampus. The effects of CSDS and agomelatine treatment on hippocampal *Bdnf* gene expression were determined after 3 weeks of agomelatine (50 mg/kg/day, i.p.) treatment following 10 days of CSDS. CSDS and chronic agomelatine treatment did not induce any change in total *Bdnf* and *Bdnf* exon VI mRNA expression. However, *Bdnf* exon IV gene expression was significantly reduced in stressed-HEC mice, but the level of *Bdnf* exon IV mRNA was not different between control-HEC and stressed-AGO mice. *p < 0.05, one-way ANOVA followed by Newman-Keuls multiple comparisons test. Each result is expressed as the mean ± S.E.M. of n = 21 (control mice), 12 (stressed HEC mice) and 12 (stressed AGO mice). AGO, agomelatine; HEC, hydroxyethylcellulose.

mRNA expression of total *Bdnf* and *Bdnf* exons IV and VI was quantified in the hippocampus of control-HEC, stressed-HEC and stressed-AGO mice. As shown in Fig. 5, while a one-way ANOVA showed that CSDS and agomelatine treatment failed to induce any expresion modification in total *Bdnf* [F(2,44) = 0.4390, p = 0.6474] and *Bdnf* exon VI [F(2,43) = 0.9738, p = 0.3858] gene expression, the same analyze followed by Newman-Keuls multiple comparisons test showed that *Bdnf* exon IV mRNA level was significantly reduced in CSDS mice, compared to non-stressed mice [F(2,43) = 4.871, p < 0.01]. Interestingly, stressed-AGO mouse *Bdnf* exon IV mRNA level did not differ from that of control-HEC [F(2,43) = 1.767, p > 0.05].

### Effect of CSDS and chronic agomelatine treatment on synaptic plasticity markers mRNA expression

A one-way ANOVA showed no difference between the three groups for *PSD-95, Synaptophysin, Spinophilin* and *Synpasin I* gene expression [F(2,39) = 2.534, p = 0.0923; F(2,39) = 2.528, p = 0.0928; F(2,39) = 0.06687, p = 0.9354 and F(2,39) = 0.487, p = 0.6181, respectively] (Table 2). In contrast, a one-way ANOVA followed by Newman-Keuls multiple comparisons test showed that *MAP-2* hippocampal mRNA level was decreased by CSDS in HEC-treated mice [F(2,37) = 3.538, p < 0.05], and that this effect was prevented by chronic agomelatine administration [F(2,37) = 3.555, p < 0.05].

### Effect of CSDS and chronic agomelatine treatment on epigenetic modifying enzymes mRNA expression

A one-way ANOVA showed that gene expression of histone acetyltransferase *MYST2* was not modified in any of the groups [F(2,39) = 0.3391, p = 0.7145] (Table 3). In contrast, CSDS decreased the expression of histone deacetylases *HDAC2* [F(2,37) = 4.534, p < 0.05, one-way ANOVA followed by Newman-Keuls multiple comparisons test] and *HDAC6* mRNA (p < 0.05, Kruskal-Wallis test followed by Dunn’s multiple comparisons test). Interestingly, this effect was prevented by chronic agomelatine treatment [*HDAC2*: F(2,37) = 3.249, p < 0.05 and *HDAC6*: p < 0.05, respectively]. At histone methylation level, a one-way ANOVA followed by Newman-Keuls multiple comparisons test indicated that CSDS reduced the expression of the histone methyltransferase *MLL3* [F(2,37) = 3.661, p < 0.05]. This reduction could also be reversed by chronic agomelatine treatment [F(2,37) = 3.082, p < 0.05]. However, the mRNA expression of neither *KDM5B*, an histone lysine demethylase [F(2,39) = 0.2727, p = 0.7628], nor *DNMT3B*, a DNA methyltransferase [F(2,38) = 0.3608, p = 0.6995], nor *GADD45B* mRNA, a DNA demethylation-associated protein [F(2,37) = 0.4995, p = 0.6109] was modified by CSDS or agomelatine treatment (Table 3).

## Discussion

The effects of chronic social defeat stress and chronic agomelatine treatment on mouse behavior and plasticity gene expression reported here showed that agomelatine failed to reverse stress-induced alterations on anxiety- and social-like behaviors, but was able to prevent CSDS deleterious effects on cognitive performances and hippocampal gene expression.

Krishnan *et al*.^10^ initially demonstrated that a population of C57BL/6J subjected to the CSDS paradigm could be separated into susceptible mice, with behavioral and molecular alterations, and resilient mice that were less prone to develop such deficits. Those two groups could be separated using social avoidance test, based on the natural propensity of mice to interact with unfamiliar mouse^34^. Here, the social avoidance test, performed 24 h after the end of the last defeat session, revealed that 59% of total mice subjected to the stress protocol were susceptible and 41% were resilient, as expected from data in the literature^10^. We assessed the effect of chronic agomelatine treatment on stress-induced behavior and molecular modifications on susceptible mice only. To this aim, social avoidance test was replicated on D29, after a 3-week treatment with either agomelatine or its vehicle in CSDS-exposed mice, or vehicle in control mice. However, after this 3-week treatment delay, no statistical difference in the social avoidance test could be found between the 3 groups of mice. The loss of the stress effect, which was clearly present on D11, could result from a marked decrease in the interaction ratio mean of control group on D29 (−42%). According to previous work^9^, mice habituation or disinterest to arena exploration is unlikely to explain this behavior. Animal isolation^11^ and daily repeated vehicle injections^35^ which are known to be meaningful stressors may have contributed to this confounding effect. However, the fact that the interaction ratio of CSDS-exposed mice treated with agomelatine did not differ from that of vehicle-treated mice suggest that this antidepressant was ineffective to reverse a depression-related behavior in the present social avoidance test. Those results did not meet previous results since agomelatine after chronic administration at the same dose ranges has been shown to display significant efficacy in various preclinical depression-like models related to stress including the transgenic GR-i mouse^36^,^37^, the corticosterone-treated mouse^38^, the chronic mild stress in rats^39^ and the prenatally stressed rat^29^,^40^, but for most of them, the stress procedure was different and based on much milder stressors than those applied here. As expected from CSDS experiments, susceptible mice displayed a strong decreased in the exploration of the center of an open field on D11, indicating a clear-cut anxious-like phenotype^41^,^42^. On D29, the effect of social defeat stress on anxiety-related behavior was still present in stressed-HEC mice, but again, agomelatine was not able to reverse this behavior. These data are not in line with previous results since agomelatine at antidepressant doses was shown to possess anxiolytic properties in naïve rats^43^,^44^ and also in various rodent depression-like models^37^,^38^,^40^. In particular, in the open field, a chronic agomelatine treatment was shown to reverse the anxiety-like phenotype induced by chronic corticosterone consumption in drinking water^38^. It was also demonstrated that agomelatine administered either acutely or sub-chronically was able to reduce anxiety-like behaviors of rats after a social defeat, an effect that needs the integrity of the suprachiasmathic nucleus^45^. Again, and as already suggested in the social avoidance test, CSDS is known to be very stressful^3^,^46^ and to induce stronger anxiety-related deficits than other types of stress such as chronic mild stress^11^. Agomelatine, which positive effects have been observed in mild stress paradigms, was not able to reverse the behavioral deficits induced by CSDS, in contrast to antidepressants of different families such as imipramine or fluoxetine^9^,^47^,^48^.

However, and although it had no significant efficacy on anxiety- and depressive-like behaviors in the present study, agomelatine showed clear cut effects on CSDS-induced memory impairments measured with the NOR test, a well-validated paradigm for evaluating episodic memory in rodents^49^. When testing the mice on D30, no deficit in the short-term memory of CSDS-subjected animals was observed in ITI1 conditions, but the memory performances of stressed-HEC mice were significantly altered in the ITI24 ones. These data corroborated previous studies that also described impaired cognition in social-defeated mice using the NOR test^16^,^41^ with mnesic alterations in that case recorded at both short and long term, but measured 5 days only after the last defeat session. Interestingly, we showed that long term memory was still affected 3 weeks after CSDS. In addition, chronic agomelatine treatment reversed CSDS-induced cognitive dysfunction in this behavior, as previously demonstrated in various memory-like models. Indeed, agomelatine was shown to produce memory facilitating effects in the NOR task in naïve rats^25^,^26^ or to improve mnesic alteration in mice exposed to unpredictable mild stress^27^. Agomelatine was also effective in enhancing animal performances in other cognition tests such as the radial-arm water maze^28^ and the Morris water maze^27^ and it improves social memory in prenatal stressed rats^29^. The positive profile of agomelatine on memory processes makes it stands out from classical antidepressant drugs, for which the effect on memory is still matter of debate (for exhaustive review see ref. 5).

The involvement of the neurotrophic factor BDNF in mnesic processes has been clearly demonstrated^50^,^51^. Lesion studies and inactivation models have shown that in the NOR test object recognition memory was hippocampus-dependent^52^,^53^ when using long ITI (24 h), as we did presently, and not a short one, further supporting a delay-dependent role for the hippocampus in object recognition memory. Therefore, we quantified mRNA levels of *Bdnf* gene in this structure, and also two of its exons which have been shown to be reduced in CSDS, the *Bdnf* exons IV and VI^47^. In our conditions, CSDS failed to induce any modification in total *Bdnf* gene expression. This does not meet previous results that demonstrated a clear downregulation of total *Bdnf* gene in the hippocampus of social defeated mice, at both short and long term^47^,^54^,^55^,^56^,^57^, although one study reported no change in the same conditions^58^. While *Bdnf* exon VI mRNA gene expression was also unchanged, *Bdnf* exon IV mRNA was significantly downregulated in CSDS stressed mice. Interestingly, this effect was abolished after a 3-week treatment with agomelatine. These data could be related to those published using imipramine, where a 10 day social defeat stress paradigm downregulated hippocampal *Bdnf* exon IV and a 4-week imipramine treatment restored basal mRNA level^47^.

Previous studies had also demonstrated the enhancing effect of chronic agomelatine (40–50 mg/kg i.p.) on hippocampal *Bdnf* gene expression both in naïve rodents^26^,^59^,^60^,^61^ and in depressive like-models^27^,^37^. The particular regulation of the *Bdnf IV* transcript had already been proposed to be crucial in the mechanism of action of antidepressant drugs^37^. Interestingly, this transcript is known to selectively stimulate proximal dendrites growth in primary cultures of rat hippocampal neurons^62^. Therefore, it can be proposed that in CSDS, the decrease in *Bdnf* exon IV gene expression might led to a reduced synaptic plasticity leading to the cognitive impairments observed in the NOR test.

To further evaluate this hypothesis, we then quantified hippocampal mRNA levels of synaptic plasticity markers, especially those of genes that have already been associated with cognitive alterations^63^,^64^,^65^. While *PSD-95, Synaptophysin, Spinophilin* and *Synapsin I* mRNA levels remained unchanged whatever the conditions, CSDS decreased *MAP-2* gene expression and this effect was prevented by agomelatine treatment. MAP-2 is a protein that regulates microtubules and dendritic remodeling^26^. Long-term reduced hippocampal expression of the latter gene has been previously described in mice subjected to stress protocols^66^, but to our knowledge, this is the first study revealing social stress-induced *MAP-2* gene expression modifications. Ladurelle *et al*.^26^ have shown that a 22-day agomelatine treatment was able to strongly increase the expression of MAP-2 protein in the hippocampus of naïve rats. These last data supported our present results that showed that chronic agomelatine administration also prevented the alterations on *MAP-2* gene expression induced by CSDS, although in our conditions, changes in *MAP-2* gene expression induced by CSDS are rather mild, and should be supported by further morphological studies, to address its functional relevance. However, those changes suggested that social stress-induced downregulation of *MAP-2* along with *Bdnf* exon IV might has provoked dendritic or synaptic alterations, leading to cognitive impairments and that chronic agomelatine could exert a protective effect on mice mnemonic functions by preventing *Bdnf IV* and *MAP-2* gene expression deficits.

Whether the alterations in *Bdnf* and *MAP-2* gene expression were supported by alterations in epigenetic regulations was assessed by studying hippocampal gene expression of various epigenetic modifying enzymes. RT-qPCR experiments revealed a downregulation of *HDAC2*, and *HDAC6* gene expression in vehicle-treated CSDS mice. Interestingly, chronic agomelatine reversed these alterations. Since these epigenetic markers are a marker of active transcription^67^, it appears that they were probably not related to the downregulation of *Bdnf* exon IV and *MAP-2* expression observed after CSDS. However, the effect of agomelatine on *HDAC2* mRNA is consistent with the work of Boulle *et al*.^30^, wherein a 25-day agomelatine treatment (50 mg/kg/d i.p.) was able to prevent hippocampal *HDAC2* downregulation observed in C57BL/6J mice exposed to chronic ultra mild stress, but had no effect by itself in non-stressed mice. Moreover, *HDAC2* expression downregulation in the hippocampus was also observed just after a chronic restraint stress^68^, in a maternal separation model^69^ and in a genetic model of depression based on impaired glucocorticoid receptor expression^70^, suggesting that *HDAC2* alteration is a long lasting phenomenon. In CSDS, Covington *et al*.^71^ also evidenced a reduced HDAC2 mRNA and protein levels in the nucleus accumbens 24 h and 2 weeks after the last defeat episode. *HDAC6* mRNA level has already been shown to be decreased in the dorsal raphe of CSDS-exposed mice^72^, but to our knowledge, our study is the first which demonstrated such regulation in the hippocampus. In addition, we also described a stress-induced modification in the expression of *MLL3*. MLL3 is a histone H3K4-specific methyltransferase^73^, and hypermethylation at this site is known to have a positive impact on gene transcription^74^,^75^. Consequently, a reduced *MLL3* mRNA level in this structure might lead to a general decrease in histone H3K4 methylation, therefore inhibiting gene transcription, which could explain CSDS-induced *Bdnf* exon IV and *MAP-2* downregulation. Chronic agomelatine, normalizing *MLL3* mRNA level, could prevent the deleterious stress effect on the latter gene expression. Nevertheless, no formal link can be found in the literature between *MML3* and these two genes. Gupta *et al*. showed that in rats, memory formation was associated with increased global hippocampal H3K4 methylation^76^ and data suggested that this hypermethylation was set at *Bdnf* exon I, but not exon IV, promoter. However, it is interesting to note that *MLL3* has also been involved in genome-scale circadian transcription^77^ further linking the antidepressant efficacy of agomelatine to its circadian effect.

In conclusion, we showed that agomelatine could reverse the cognitive and gene-related effects of social defeat stress in mice. In our conditions, CSDS induced long lasting deleterious effects on C57BL/6J social behavior, anxiety- and memory-related behaviors as well as hippocampal gene expression. While chronic agomelatine treatment was ineffective in social and anxiety-like behaviors, it significantly reversed the memory impairments observed in the NOR test. These data provide evidences that agomelatine could be a relevant therapy to alleviate depression-associated cognitive disorders^23^. CSDS-induced mnesic deficits might be related to *Bdnf* exon IV and *MAP-2* gene expression alteration. Chronic agomelatine prevented these effects, possibly in part through epigenetic-related mechanisms. However, further studies are needed to evaluate the direct impact of CSDS and agomelatine on epigenetic marks and homeostasis at the *Bdnf* exon IV promoter and to clarify through which mechanisms this compounds could achieve its effects.

## Additional Information

**How to cite this article:** Martin, V. *et al*. Effect of agomelatine on memory deficits and hippocampal gene expression induced by chronic social defeat stress in mice. *Sci. Rep.* **7**, 45907; doi: 10.1038/srep45907 (2017).

**Publisher's note:** Springer Nature remains neutral with regard to jurisdictional claims in published maps and institutional affiliations.

## Supplementary Material

Supplementary Data

## Figures and Tables

**Table 1:** Primer sequences used for mRNA expression analyses by RT-qPCR with SYBR Green technology.

Genes	3′ primers	5′ primers
*Total BDNF*	AAAACCATAAGGACGCGGAC	TAGACATGTTTGCGGCATCC
*BDNF IV*	CTCTGCCTAGATCAAATGGAGCT	GAAGTGTACAAGTCCGCGTCCTT
*BDNF VI*	GCTGGCTGTCGCACGGTTCCCATT	GAAGTGTACAAGTCCGCGTCCTT
*MYST2*	GGTACTGCTCCGATACCTGC	TCTGAAGGGCTTGCTTGCAGAGTC
*HDAC2*	AGAAGATTGTCCGGTGTTTGATG	CACAGCCCCAGCAACTGAA
*HDAC6*	CAGAGCCCACCCTCAAAGAG	TCCAGGGACAGAATCAACTTGCCT
*MLL3*	TCCCGATAGTTTCGTCCCCT	AGAATGGGGATGAGGGGG
*KDM5B*	GTTTGGCAGTGGCTTTCCTG	ATGCCCACATACAACCAGGG
*DNMT3B*	GATGAGGAGAGCCGAGAACG	CAGAGCCCACCCTCAAAGAG
*GADD45B*	GTTCTGCTGCGACAATGACA	TTGGCTTTTCCAGGAATCTG
*PSD-95*	TCTGTGCGAGAGGTAGCAGA	AAGCACTCCGTGAACTCCTG
*Synaptophysin*	TCTTTGTCACCGTGGCTGTGTT	TCCCTCAGTTCCTTGCATGTGT
*Spinophilin*	AAGGCGGCCCACCATAA	GCCCATCTGCAGGAACATACTT
*Synapsin I*	CACCGACTGGGCAAAATACT	TCCGAAGAACTTCCATGTCC
*MAP-2*	TCAGGAGACAGGGAGGAGAA	GTGTGGAGGTGCCACTTTTT
*β-actin*	CCACCATGTACCCAGGCATT	CGGACTCATCGTACTCCTGC

**Table 2:** Long-lasting effect of chronic social defeat stress and chronic agomelatine on synaptic plasticity markers mRNA expression in the hippocampus.

Gene	Control HEC	Stressed HEC	Stressed AGO	Control HEC vs Stressed HEC	Stressed HEC vs Stressed AGO
*PSD-95*	1.003 ± 0.018	0.9500 ± 0.022	1.019 ± 0.025	n.s	n.s
*Synaptophysin*	1.011 ± 0.038	0.8867 ± 0.048	0.9367 ± 0.035	n.s	n.s
*Spinophilin*	1.007 ± 0.029	1.020 ± 0.044	1.023 ± 0.031	n.s	n.s
*Synapsin I*	1.003 ± 0.019	0.9750 ± 0.029	1.008 ± 0.026	n.s	n.s
*MAP-2*	1.003 ± 0.019	0.9236 ± 0.022	1.017 ± 0.337	*	#

The effects of CSDS and agomelatine treatment on hippocampal gene expression of synaptic plasticity markers were tested after 3 weeks of agomelatine (50 mg/kg/day, i.p.) treatment following 10 days of CSDS.

Neither CSDS nor chronic agomelatine modified *PSD-95, Synaptophysin, Spinophilin* and *Synpasin I* gene expression. However, mRNA expression of *MAP-2* was decreased in stressed mice treated with vehicle compared with controls and stressed mice treated with agomelatine.

*p < 0.05 “Control HEC” vs “Stressed HEC”, ^#^p < 0.05 “Stressed HEC” vs “Stressed AGO”, one-way ANOVA followed by Newman-Keuls multiple comparisons test.

Each result is expressed as the mean ± S.E.M. of n = 18 (control mice), 11 (stressed HEC mice) and 11 (stressed AGO mice).

AGO, agomelatine; HEC, hydroxyethylcellulose.

**Table 3:** Long-lasting effect of chronic social defeat stress and chronic agomelatine on histone acetylation/methylation and DNA methylation modifying enzymes mRNA expression in the hippocampus.

Gene	Control HEC	Stressed HEC	Stressed AGO	Control HEC vs Stressed HEC	Stressed HEC vs Stressed AGO
*MYST2*	1.006 ± 0.025	0.9745 ± 0.033	0.9891 ± 0.023	n.s	n.s
*HDAC2*	1.007 ± 0.027	0.8800 ± 0.023	0.9875 ± 0.031	*	#
*HDAC6*	1.010 ± 0.033	0.8833 ± 0.019	1.008 ± 0.0196	*	#
*MLL3*	1.022 ± 0.048	0.8080 ± 0.044	1.009 ± 0.078	*	#
*KDM5B*	1.003 ± 0.021	1.022 ± 0.022	0.9975 ± 0.022	n.s	n.s
*DNMT3B*	1.010 ± 0.034	1.048 ± 0.032	1.038 ± 0.033	n.s	n.s
*GADD*	1.0221 ± 0.051	0.9489 ± 0.036	1.028 ± 0.061	n.s	n.s

The effects of CSDS and agomelatine treatment on hippocampal gene expression of enzymes known to modify histone acetylation and methylation as well as DNA methylation were tested after 3 weeks of agomelatine (50 mg/kg/day, i.p.) treatment following 10 days of CSDS.

No significant differences were found between the three groups for *MYST2, KDM5B, DNMT3B and GADD45B* mRNA expression. However, concerning *HDAC2, HDAC6* and *MLL3*, stressed mice chronically treated with the vehicle showed reduced gene expression, and this effect was reversed by chronic agomelatine treatment.

*p < 0.05 “Control HEC” vs “Stressed HEC”, ^#^p < 0.05 “Stressed HEC” vs “Stressed AGO”, one-way ANOVA followed by Newman-Keuls multiple comparisons test (*HDAC2* and *MLL3*) or Kruskal-Wallis test followed by Dunn’s multiple comparisons test (*HDAC6)*.

Each result is expressed as the mean ± S.E.M. of n = 19 (control mice), 10 (stressed HEC mice) and 10 (stressed AGO mice).

AGO, agomelatine; HEC, hydroxyethylcellulose.
