# Supplementary material for: Effect of agomelatine on memory deficits and hippocampal gene expression induced by chronic social defeat stress in mice
Source: Sci Rep. 2017 Apr 4;7:45907. doi: 10.1038/srep45907 (PMC5379201; doi:10.1038/srep45907)
Supplement: Supplementary Data [file srep45907-s1.pdf]

Effect of agomelatine on memory deficits and hippocampal gene expression induced by chronic social defeat stress in mice

Vincent Martin[1], Najib Allaïli[2], Marine Euvrard[1], Tevrasamy Marday[1], Armance Riffaud[1], Bernard Franc[1], Elisabeth Mocaër[4], Cecilia Gabriel[4], Philippe Fossati[3], Stéphane Lehericy[2] and Laurence Lanfumey[1*]

[1]Centre de Psychiatrie et Neurosciences, INSERM UMR 894, Université Paris Descartes - Paris, France
[2]Centre de NeuroImagerie de Recherche - CENIR- - Inserm UMR1127- CNRS 7225, Institut Cerveau Moelle - ICM, Sorbonne Universités- UPMC UMR S 1127 - Paris, France
[3]Social and Affective Neuroscience - SAN Laboratory - Inserm U 1127- CNRS UMR 7225- Institut du Cerveau et de la Moelle- ICM - Sorbonne Universités- UPMC UMR S 1127, - Paris, France
[4]Institut de Recherches Internationales Servier, IRIS, Suresnes, France

	Change in body weight from day 1 (g)			
	**Day 11**	**Day 18**	**Day 25**	**Day 32**
Control	1.54 ± 0.30	2.13 ± 0.21	2.72 ± 0.20	2.41 ± 0.19
Stressed HEC	1.24 ± 0.51	2.48 ± 0.31	3.5 ± 0.27	2.84 ± 0.28
Stressed AGO	0.68 ± 0.31	1.84 ± 0.44	2.43 ± 0.40	1.82 ± 0.35

Table S1. Effect of chronic social defeat stress and chronic agomelatine on mice body weight gain

Each bar is the mean ± S.E.M. of n = 19 (control mice), 11 (stressed HEC mice) and 11 (stressed AGO mice). AGO, agomelatine; HEC, hydroxyethylcellulose.

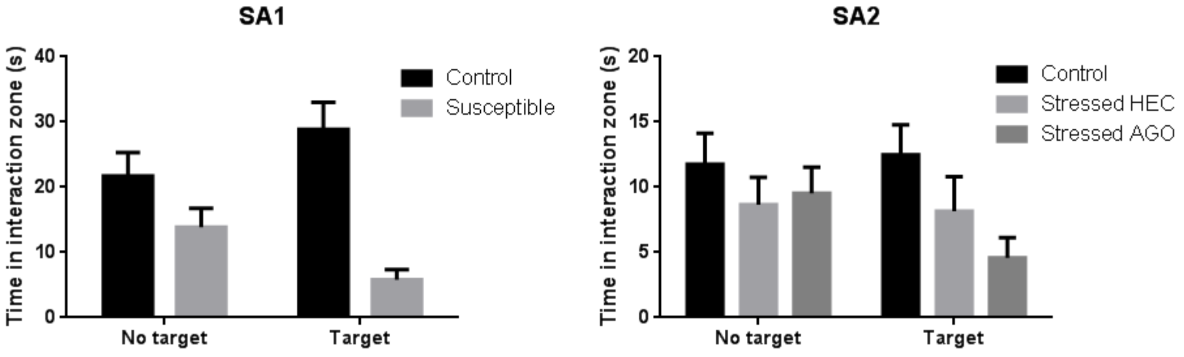

Figure S1. Time of interaction with and without target at day 11 (SA1) and day 29 (SA2).

Time spent in the virtual interaction zone of the open field was scored using a video tracking system during 150 s sessions in which the mouse was either alone (no target) or in presence of a CD1 mouse (target) in a circular wire cage.

Note that in SA1, control mice increased the interaction time with the target whether stressed susceptible mice decreased it. In SA2, the effect of stress was still present.

Each bar is the mean ± S.E.M. of n = 19 (control mice), 24 (susceptible), 11 (stressed HEC mice) and 11 (stressed AGO mice). AGO, agomelatine; HEC, hydroxyethylcellulose.SA, Social Avoidance test.
